# Mitochondrial Genome Assembly and Comparative Analysis of *Chionanthus Retusus* (Oleaceae)

**DOI:** 10.3390/genes15121523

**Published:** 2024-11-27

**Authors:** Shasha Zhai, Furong Lin, Xiuge Shu, Hongyun Niu, Qi Jing, Lei Gao, Xiangbin Gao, Dan Liu

**Affiliations:** 1 College of Agriculture and Biology, Liaocheng University, Liaocheng 252000, China; 18563510688@163.com (S.Z.); 17658258675@163.com (L.G.); 2 State Key Laboratory of Tree Genetics and Breeding, Key Laboratory of Tree Breeding and Cultivation of State Forestry and Grassland Administration, Research Institute of Forestry, Chinese Academy of Forestry, Beijing 100091, China; linfr@caf.ac.cn; 3 Shandong Academy of Forestry Sciences, Jinan 250014, China; hzkzq@163.com; 4 Shandong Aviation Emergency Rescue Center, Jinan 250014, China; 15966069344@163.com; 5 Shandong Provincial Center of Forest and Grass Germplasm Resources, Jinan 250102, China; jqgzh163@163.com

**Keywords:** *chionanthus retusus*, mitochondrial genome, phylogenetic analysis, migration sequence

## Abstract

**Background/Objectives**: *Chionanthus retusus* Lindl. & Paxton is an ornamental tree species native to North China. Research on the mitochondrial genome can elucidate the evolution and biological characteristics of *C. retusus* and better protect this important species. **Methods and Results**: This work aimed to clarify the evolutionary and phylogenetic links by sequencing, assembling, annotating, and analyzing the entire mitochondrial genome of *C. retusus*. The single-loop structure that made up the mitochondrial genome had a total length of 657,640 bp and a GC content of 44.52%. In total, 37 unique protein-coding genes, 20 tRNA genes, and 3 rRNA genes were identified. Numerous repeat sequences and migrating fragments of chloroplast sequences were found. Using the mitochondrial protein-coding genes to construct evolutionary trees, it was found that the closest relative of *C. retusus* is *C. rupicola* (Lingelsh.) Kiew. **Conclusions**: This research represents the first comprehensive set of data on the mitochondrial genome of an ancient (>500 yr) *C. retusus* specimen. In addition to elucidating the biological characteristics of *C. retusus*. The findings contribute to the Oleaceae mitochondrial genome database and offer valuable insights for future studies in molecular breeding, evolutionary biology, and genetic diversity conservation.

## 1. Introduction

*C. retusus* (Oleaceae), a deciduous shrub or tree native to China, Japan, and the Korean Peninsula, is resistant to drought, salt, and flooding [1]. In North China, it is called “April snow” due to its profuse blossoms in April [2]. The species has long been cultivated in China as an ancient and highly prized ornamental garden plant [3]. As well, the bark, roots, and leaves of *C. retusus* are used in traditional medicine, and the plant is recognized as a medicinal plant of economic value [4]. The research focus on the species, both in China and abroad, has been largely propagation technology [5,6,7], chemical composition, and medicinal value [8,9], as well as stress tolerance [10], seed dormancy characteristics [11,12], germplasm resource collection, and genetic diversity [13,14,15].

At present, research on native *C. retusus* in Shandong Province is limited to the investigation of ancient tree resources and discussions on the rescue and rejuvenation of several ancient specimens of *C. retusus* [16,17,18]. Genetic research mainly focuses on the following four aspects: the telomere-to-telomere gap-free reference genome [19], genome-wide MIKC-type MADS-box genes [20], genetic diversity and population structure of *Chionanthus virginicus* [21], and chloroplast genomes [22]. Ancient *C. retusus’* mitochondrial genome has not yet been investigated up until now. As stated in [23], mitochondrial genome research has potential applications in plant genomics, with potential applications in plant breeding and conservation. Accordingly, the goal of this work was to explore the mitochondrial genome of an ancient *C. retusus* through comparisons with other Oleaceae species. In addition to providing crucial reference data for the evolution, genetic diversity conservation, and molecular breeding of Oleaceae species, the sequencing, assembly, annotation, and analysis of the mitochondrial genome will help us comprehend the biological characteristics of *C. retusus* and offer a more thorough foundation for the preservation of ancient and famous trees in this family.

## 2. Materials and Methods

### 2.1. Plant Material and Genome Sequencing

The plant material sequenced in this research was obtained from a first-grade ancient (>500 yr) *C. retusus*. In September 2023, fresh young leaves that were whole, mature, and free of pests and disease of the millennium ancient *C. retusus* (approximately 1000 years old) growing in Zhuangyuangou Village, Yanzhuang Street, Gangcheng District, Jinan City, Shandong Province (36°7′37″ N, 117°42′51″ E, altitude 250 m) were collected. The samples were kept in a refrigerator set to −80 °C after being rapidly frozen in liquid nitrogen. Total DNA was obtained by following the steps of the Tiangen kit, and the original sequence data was obtained by sequencing and library construction using the nanopore GridION sequencing platform and Illumina Novaseq 6000 platform [24]; low-quality sequences (mass value of Q < 19) were deleted.

### 2.2. Genome Assembly and Annotation

Using the Flye (v2.9.2-b1786) (developed by Kolmogorov et al., available at https://github.com/fenderglass/Flye, accessed on 1 November 2023) software’s default settings, the long-read sequencing data was assembled directly, yielding graphical assembly results in the GFA format [25]. A BLAST dataset of contigs, using the FASTA file format in the makeblastdb built library, was obtained. A gene sequence from the mitochondria of *Arabidopsis* was the query sequence. Contigs were found in the *C. retusus* mitochondrial genome fragments using the following criterion: -evalue 1e-5 -outfmt 6 -max_hsps 10 -word_size 7 -task blastn-short. The GFA file was visualized using Bandage (v0.8.1) (developed by Ryan Wick et al., available at https://github.com/rrwick/Bandage, accessed on 7 November 2023), and a mitochondrial genome sketch was produced by screening mitochondrial contigs in accordance with the BLASTn results [26]. Using bwa software (v0.7.17) (developed by Heng Li, available at http://bio-bwa.sourceforge.net/, accessed on 15 November 2023), long-read and short-read data were compared to mitochondrial contigs. For later mixed assembly, the matched mitochondria’s readings were filtered, exported, and stored separately. Together with the aforementioned short-read and long-read sequencing data, a hybrid assembly technique was used to construct the mitochondrial genome of *C. retusus* [27]. Using Unicycler’s default settings, the hybrid assembly produced the mitochondrial genome of *C. retusus* [28].

Geseq software (v2.03) was performed to annotate the genomes [29], with the reference genomes being the protein-coding mitochondrial genomes of *Liriodendron tulipifera* L. (NC_021152.1) and *Arabidopsis thaliana* (L.) Heynh. (NC_037304). The genome of *C. retusus* was annotated using the mitochondrial genome annotation tool IPMGA (http://www.1kmpg.cn/ipmga/, accessed on 20 November 2023), and this tool has shown promising results for identifying splice sites and trans-splicing of genes in angiosperms. tRNAscan-SE software (v.2.0.11) (http://lowelab.ucsc.edu/tRNAscan-SE/, accessed on 20 November 2023) was used to annotate the mitochondrial genome’s tRNA [30], while BLASTN software (v2.13.0) (https://blast.ncbi.nlm.nih.gov/Blast.cgi, accessed on 20 November 2023) was used to annotate the rRNA [31]. The mitochondrial genome’s annotation errors were manually fixed using the Apollo software (v1.11.8) (https://github.com/GMOD/Apollo, accessed on 25 December 2023) [32]. The final annotated mitogenome was added to GenBank with accession number PQ072898.

### 2.3. Analysis of RSCU and RNA Editing Sites Prediction

Phylosuite software (v1.1.16) (https://dongzhang0725.github.io/, accessed on 1 December 2023) was used to extract the protein-coding gene (PCG) sequences [33]. The PCGs in the mitochondrial genome were subjected to a codon preference analysis using Mega software (v7.0) (https://www.megasoftware.net/, accessed on 1 December 2023), and the genome’s codon preference was analyzed by calculating values of relative synonymous codon usage (RSCU) [34].

Using the sequences of all PCGs represented by the *C. retusus* specimen’s mitochondrial genome as input files, Deepred-mt was used to predict the C-to-U RNA editing sites of mitochondrial PCGs [35]. The convolutional neural network (CNN) model serves as the foundation for the tool. Compared to earlier prediction tools, its accuracy is great. Results were retained if their probability values were higher than 0.9.

### 2.4. Analysis of Sequence Repeats

DNA sequence repetitions, such as tandem repeats, dispersed repeats, and microsatellite sequences, were identified using the MISA (v2.1) [36], TRF (v4.09) [37], and REPuter [38] web servers. Excel (2021) software and the Circos package (v0.69.9) were used to visualize the results [39].

### 2.5. Chloroplast to Mitochondrion DNA Transformation

To better analyze the sequence migration of the specimen of ancient *C. retusus*, GetOrganelle software (v1.7.7.0) (https://github.com/Kinggerm/GetOrganelle, accessed on 7 December 2023) was used to assemble the chloroplast genome [40]. Chloroplast genome annotation was done using CPGAVAS2 [41], and CPGView was used to correct the annotated chloroplast genome [42]. After that, homologous segments were analyzed using BLASTN software (v2.13.0) [31], and the Circos package (v0.69.9) was used to visualize the findings [39].

### 2.6. Synteny and Phylogenetic Analysis

The BLASTN results of the mated comparisons of individual mitochondrial genomes, as obtained from the BLASTN process, as well as homologous sequences over 500 bp in length, were retained to obtain the Multiple Synteny plots as conserved collinear blocks.

Based on kinship, closely related species were selected, and their mitochondrial genomes were downloaded. Shared genes were extracted using PhyloSuite software (v1.1.16) [33], and MAFFT software (v7.505) (https://mafft.cbrc.jp/alignment/software/, accessed on 15 December 2023) [43] was used to conduct multiple sequence alignment analysis. IQ-TREE software (v1.6.12) (https://github.com/iqtree/iqtree2, accessed on 15 December 2023) [44] was utilized for phylogenetic analysis, and ITOL software (v6) (https://itol.embl.de/, accessed on 15 December 2025) was employed to show the phylogenetic analysis findings [45].

## 3. Results

### 3.1. C. Retusus Mitochondrial Genome Characteristics

**Figure 1:**
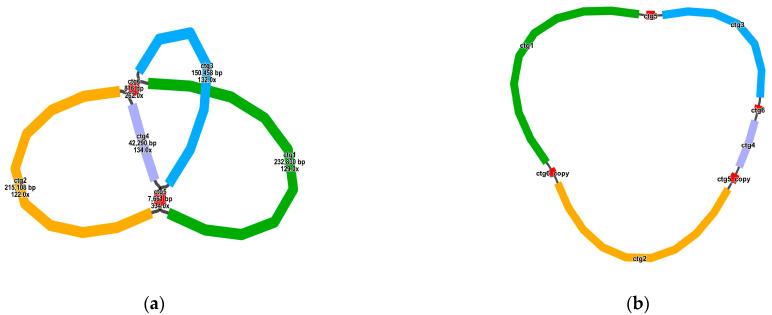
Schematic representation of the Flye assembly results. (**a**) We used six nodes to represent the six contigs obtained from the assembly, named ctg1 through ctg6, with the names labeled on the nodes. Sequencing depth and length information for each node are also annotated. Black lines are used to connect nodes where overlapping regions exist, indicating that the corresponding contigs can be merged into a longer sequence. The six contigs and their linkages represent the complete mitochondrial genome. (**b**) Based on the linkages between the nodes, we constructed a circular genome in the following order: ctg1-ctg5-ctg3-ctg6-ctg4-ctg5_copy-ctg2-ctg6_copy, representing a classical circular double-stranded DNA structure.

We assembled the raw sequencing data using the Flye software, resulting in six overlapping contigs (Table S1). These six contigs represent the complete mitochondrial genome sequence. Different colored nodes (ctg1–ctg6) were used to represent these contigs, with black connecting lines indicating overlaps between the nodes. Figure 1a shows the lengths, sequencing depths, and linkages of these six contigs. Considering the sequencing depth, nodes ctg5 and ctg6 are potential repeat regions that may occur twice in the genome. Based on the long-read sequences, we determined that the mitochondrial genome forms a circular structure consisting of nodes arranged in the order of ctg1-ctg5-ctg3-ctg6-ctg4-ctg5_copy-ctg2-ctg6_copy (Figure 1b), representing a classical circular double-stranded DNA structure. In addition, two pairs of repeats may mediate potential recombination constructs, and the genome has a variety of different configurations (Figure S1).

**Figure 2:**
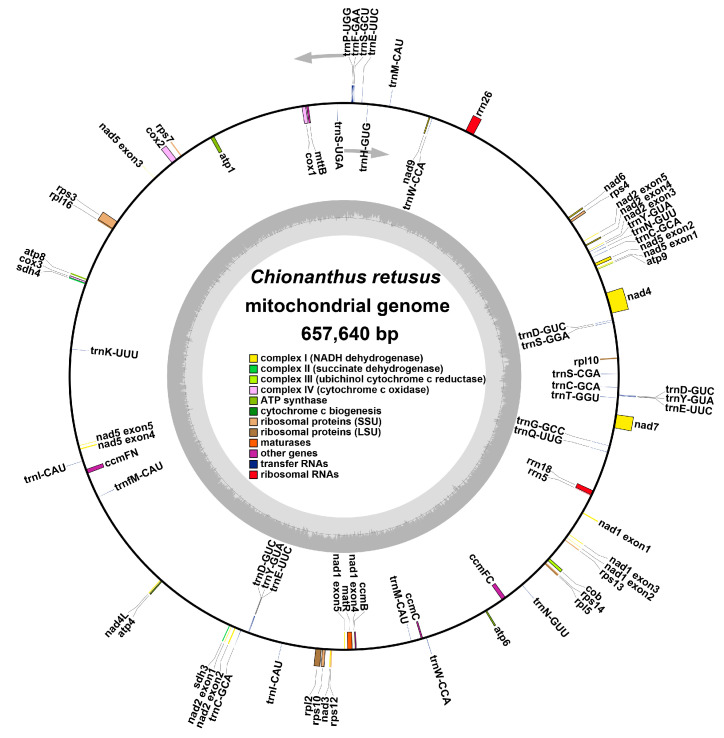
Annotation results of *C. retusus* mitochondrial genome and genome map.

Annotation of the mitochondrial genome of *C. retusus* revealed 37 unique protein-coding genes (including 24 unique mitochondrial core and 13 non-core genes), 20 tRNA genes, and three rRNA genes (Table 1, Figure 2). The core genes include five ATP synthase genes, nine NADH dehydrogenase genes, four cytochrome C biogenetic genes, three cytochrome C oxidase genes, one membrane transport protein gene, one maturation enzyme gene, and one panthenol-cytochrome C reductase gene. The non-core genes include four large ribosome subunit genes, seven small ribosome subunit genes, and two succinate dehydrogenase genes.

### 3.2. Codon Usage Preference and RNA Editing Sites Prediction

**Figure 3:**
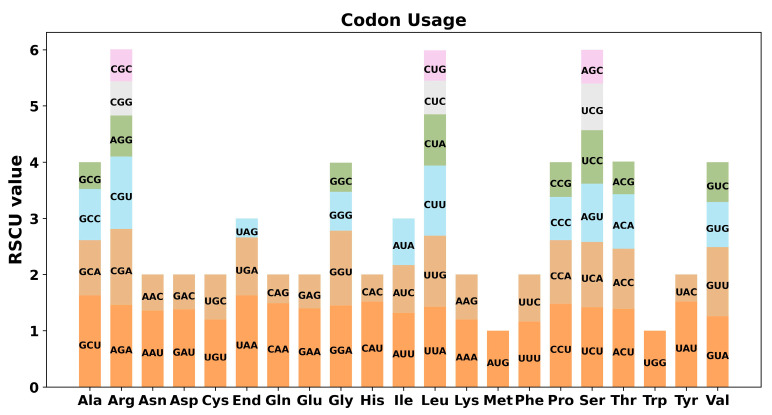
RSCU of *C. retusus* mitochondrial genome. The x-axis displays the codon family. The number of times the codon is seen in relation to the usage of the uniform synonymous codon is known as the RSCU value.

Thirty-seven unique PCGs of *C. retusus* mitochondria were subjected to codon preference analysis; the codons used for each amino acid are shown in Table S2. Amino acids were thought to favor codons with RSCU values higher than 1. As shown in Figure 3, in addition to the starting codon AUG and the RSCU value of tryptophan (UGG) being both 1, there was a general codon use preference in mitochondrial PCGs. For instance, stop codons exhibit a stronger preference for UAA and alanine (Ala) for GCU; both of these codons’ RSCU values (1.63) are highest in mitochondrial PCGs.

**Figure 4:**
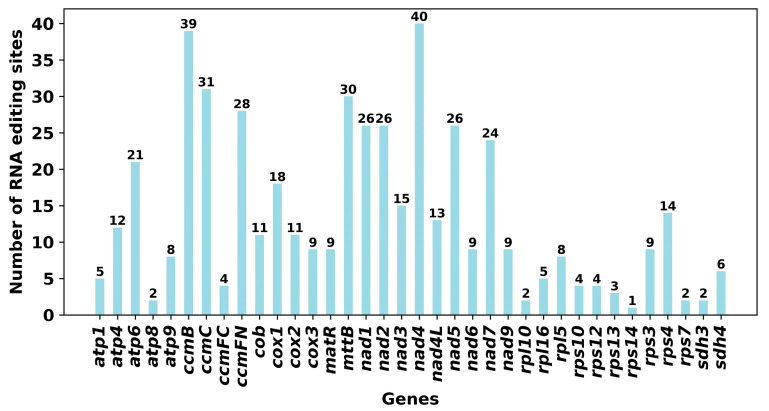
RNA editing site distribution in the mitochondrial genome of *C. retusus*. Genes that code for proteins are shown on the x-axis, while the number of RNA editing sites is shown on the y-axis.

Thirty-seven distinct PCGs from the mitochondria of *C. retusus* were found to have RNA-editing events. Base C-to-U alterations were found at all 486 latent RNA-editing sites (Figure 4). With 40 RNA editing sites, the *nad*4 gene is the most edited gene in the mitochondria, followed by the *ccm*B gene, which has 39 RNA editing events.

### 3.3. C. Retusus Mitochondrial Genome Repeat Sequence Analysis

**Figure 5:**
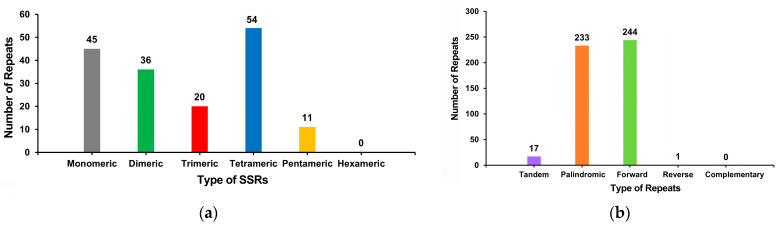
Mitochondrial genome repeat analysis of *C. retusus*. (**a**) The number of repeats (y-axis) is plotted against the SSR type (x-axis). Type of SSR is indicated by color: gray, monomer SSRs; green, dimer SSRs; red, trimer SSRs; blue, tetramer SSRs; and yellow, pentamer SSRs. No hexamer SSRs were detected in the mitochondrial genome.; (**b**) The number of repeats (y-axis) is plotted against the type of repeat sequence (x-axis), represented by different colors: purple; tandem repeats; orange, palindromic repeats; green, forward repeats; red, reverse repeats. No complementary repeats were detected in the mitochondrial genome.

The repeat sequence analysis indicated a total of 166 SSRs, of which 48.80% were in the form of monomers and dimers (Figure 5, Table S3). Of the 45 monomer SSRs, 53.33% (24) were adenine (A) monomer repeats. Seventeen tandem repetitions with lengths ranging from 9 to 29 bp and matching degrees greater than 68% were found. In the mitochondrial DNA of *C. retusus*, scattered repeats were found. As a result, 233 palindromic repeats, 244 forward repeats, and one reverse repeat were among the 478 pairs of repeats larger than or equal to 30 that were discovered. No complementary repetitions were found in the mitochondrial genome. The longest palindromic repeat and forward repeat were 7668 bp and 277 bp, respectively.

### 3.4. Chloroplast to Mitochondrion DNA Transformation of C. retusus

**Figure 6:**
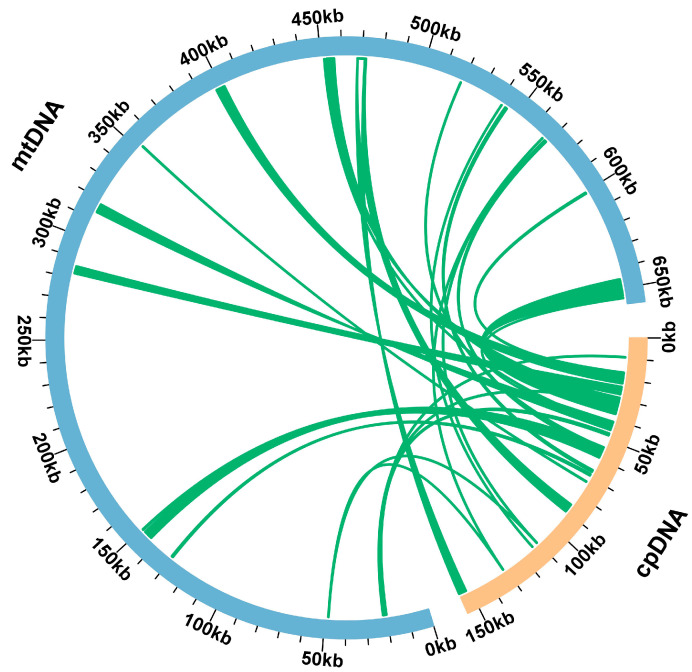
Gene migration between chloroplast and mitochondrial genomes in *C. retusus*. Blue arc: mitochondrial genome; orange arc: chloroplast genome. Homologous genomic segments are represented by the green lines that run between the arcs.

According to the sequence transformation analysis, there are 25 mitochondrial genome fragments of the *C. retusus* that are homologous to chloroplast genomes, which have a combined length of 39,288 bp, or 5.97% of the mitochondrial genome’s overall length (Figure 6). MTPT11 is the longest (8055 bp). By annotating homologous sequences, 25 complete genes—14 protein-coding genes and 11 tRNA genes—were also discovered in 25 homologous fragments.

### 3.5. Synteny and Phylogenetic Analysis of C. retusus

**Figure 7:**
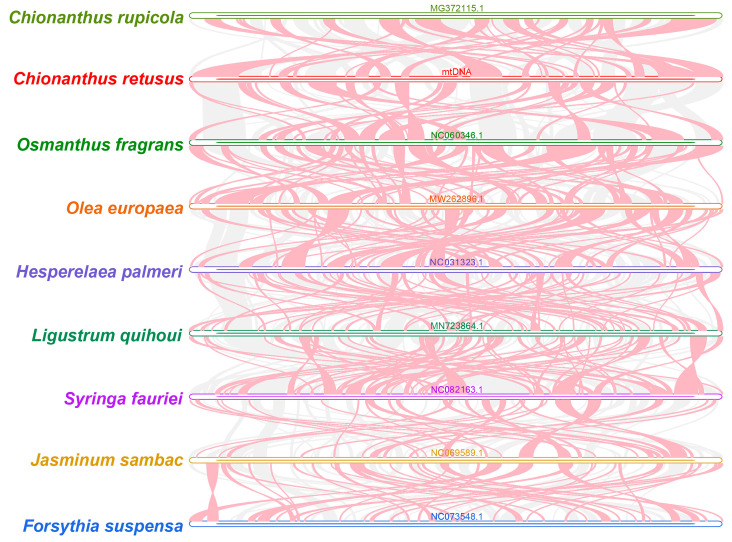
Homology of the mitochondrial genome. The gray area indicates the area with good homology, while the area where the inversion occurred is indicated by the red arc area. The length of the collinear block is not less than 0.5 kb.

A good deal of homologous collinear blocks were found in *C. retusus* and closely related species in Oleaceae (Figure 7). Though these collinear blocks were shorter in length and contained some blank regions where these sequences were specific to *C. retusus* species; that is, there was no homology with the other species. The findings showed that the collinear blocks of these nine species’ mitochondrial genomes were arranged in an inconsistent order. The mitochondrial genome of *C. retusus* evidently underwent extensive genome rearrangement compared to the genomes of close relatives; in other words, the mitochondrial genome sequences of these nine Oleaceae species are extremely unconserved in the arrangement order and have undergone frequent genome recombination.

**Figure 8:**
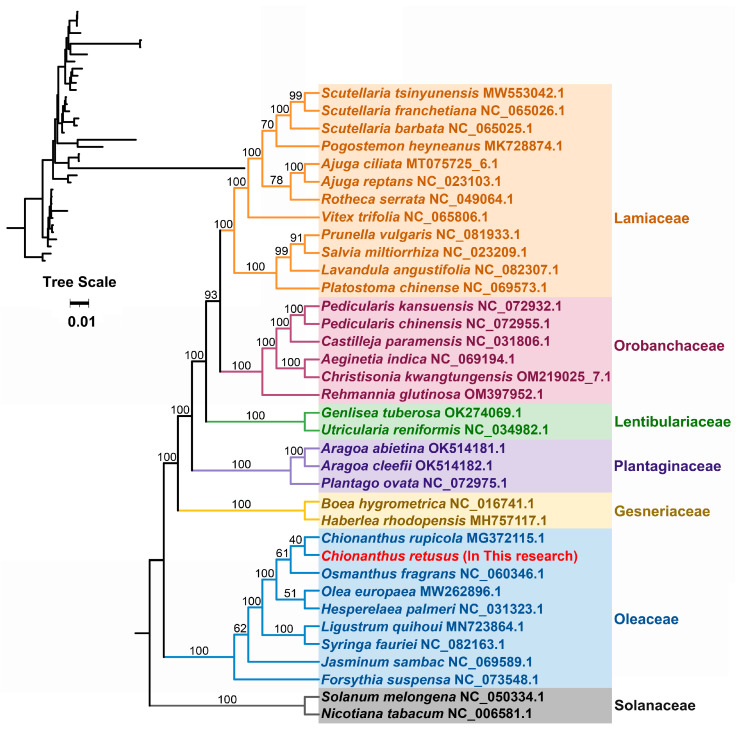
Phylogenetic analysis.

The DNA sequences of 26 conserved mitochondrial protein-coding genes—*atp*1, *atp*4, *atp*6, *atp*8, *atp*9, *ccm*B, *ccm*C, *ccm*FC, *ccm*FN, *cob*, *cox*2, *cox*3, *mat*R, *mtt*B, *nad*1, *nad*2, *nad*3, *nad*4, *nad*4L, *nad*5, *nad*6, *rpl*2, *rps*3, *rps*4, *rps*12, and *rps*13 were used to create phylogenetic trees for 36 angiosperm species under seven categories (Figure 8). Two Solanaceae mitochondrial genomes were used as outgroups. The mitochondrial DNA-based phylogenetic topology aligned with the most recent angiosperm phylogeny group classification. *C. retusus* belongs to the Oleaceae family in the order Lamiales and is the closest relative of *Chionanthus rupicola*.

## 4. Discussion

In eukaryotic cells, mitochondria are important organelles that supply energy for different cells’ physiological functions. Compared to animal cells, plant cells have a more complicated mitochondrial genome. Generally, angiosperms typically have mitochondrial genomes between 200 Kb to 3 Mb in size. These genomes vary greatly in size and structure, but they also have highly conserved genes and sparse distribution. The mitochondria also contain a large number of non-coding sequences, and there is a large amount of RNA editing [46,47].

Because of the high rate of mutation in the mitochondrial genome, this genome of the family Oleaceae has not been thoroughly researched. Wang and Zhang sequenced the mitochondrial genome sequencing of *Osmanthus fragrans* (Thunb.) Lour. in 2021. The authors reported the mitochondrial genome size was 563,202 bp, and the total GC content was 44.58% [48]. Sadder et al. sequenced the mitochondrial genome of *Olea europaea* L. in 2023, which consisted of 710,808 base pairs with a GC content of 44.7% [49]. In the same year, Song et al. analyzed the mitochondrial genome of *Forsythia suspensa* (Thunb.) Vahl, which is 535,692 bp in length, has a ring structure and a GC content of 44.90% [50]. The size of the mitochondrial genome of *C.retusus* assembled in this study was 657,640 bp, which is larger than that of *O. fragrans* and *F. suspensa*, and smaller than that of *O. europaea*. The GC content was 44.52%, which was slightly lower than that of the three Oleaceae plants. These differences—which may be caused by the expansion of gene intervals—warrant further investigation of mitochondrial genomes in Oleaceae [51].

The eukaryotic genome contains 64 codons encoding 20 different amino acids and three stop codons. Multiple codons encode every amino acid, with the exception of tryptophan and methionine. There are significant differences in the utilization rates of genomic codons among different species and organisms [52]. This preference is thought to result from the gradual formation of a relative balance within cells over an extended period of evolutionary selection. For example, in the mitochondrial genomes of *F. suspensa* and *C. retusus*, the RSCU values of tryptophan UGG (Trp) were 1. The RSCU value of the most commonly used codon AUG (Met) for *F. suspensa* is 2.98, which is much larger than the RSCU value of 1 for the starting codon AUG for *C. retusus*. In addition, *F. suspensa’*s mitochondrial genome used the codons GCU (Ala), UAU (Tyr), and CAU (His) less frequently. Among them, CUG (Met) and UUG (Met) are used least frequently [50]; in contrast, alanine (Ala) in the mitochondrial genome of *C. retusus* has a higher preference for GCU. Stop codons prefer UAA, and their RSCU values are the highest in mitochondrial PCGs (1.63). By comparison, it was found that although *F. suspensa* and *C. retusus* (both in the Oleaceae family) are closely related, there are still significant differences in their codon usage preferences.

The DNA sequence of biological cells contains many repeat sequences, which can be divided into tandem and dispersed repeats [53]. Repeat sequences in mitochondrial genomes are often essential for intermolecular recombination [54,55]. For example, 137 simple repeats (SSRs) were found in the mitochondrial genome of *F. suspensa* [50], whereas 166 SSRs were found in *C. retusus*. In the case of *F. suspensa*, monomer and dimer forms account for 54.75% (75) of the total SSRs; whereas, in *C. retusus*, monomers and dimers account for 48.80% of SSRs (5.95% less than in *F. suspensa*). Moreover, there are also tetramers and hamerers in the mitochondrial genome repeat sequence of *F. suspensa*, accounting for 27.01% (37) and 0.73% (1), respectively, of sequences that are not found in the mitochondrial genome repeat sequence of *C. retusus*. In addition, adenine (A) monucleotide repeats in *F. suspensa* account for 50% (21) of monomer SSRs, and adenine (A) monomer repeats in *C. retusus* suspensa account for 53.33% (24) of monomer SSRs. This may also be why the high monomer A content in the mitochondrial genome of *C. retusus* is consistent with the evolutionary trajectory of base composition in plant organelle genomes [56,57,58]. In general, the largest repetitions within a species (typically more than 1 kb in angiosperms) constitute recombination, resulting in isomerization [59,60]. The longest reported scattered repeat in the mitochondrial genome of an angiosperm was over 1 kb (10,578 bp) and may be the cause of heterodimerization. In contrast, the longest palindrine repeat of *C. retusus* mitochondrial genome was 7668 bp, the longest forward repeat was only 277 bp, and there were 478 pairs of repeats with a length greater than or equal to 30 bp. These findings are of great significance in terms of the potential expansion of the *C. retusus* mitochondrial genome size and degree of intermolecular combination [47,60].

Certain chloroplast fragments migrate into the mitochondrial genome during the process of mitochondrial evolution, and the length and sequence similarity of these migrating fragments vary among species [47]; the transferred fragments usually account for 1–12% of the total length of mtDNA [61]. Twenty-five homologous chloroplast genome segments, or 5.97% of the entire mitochondrial genome, were found in the *C. retusus*. In the report of *F. suspensa’*s mitochondrial genome, 25 homologous sequences were identified, accounting for about 3.93% of the total genome of mitochondria [50]. In *F. suspensa* and *C. retusus*—both species of Oleaceae—the amount of genome fragments that have migrated is comparable, but the proportion differs due to the difference in the total length of the mtDNA. Compared with *Phoenix dactylifera* L., a deciduous tree with a migration ratio of 10.3% [62], the two species of Oleaceae are relatively conserved and have low migration rates.

As gene sequencing technology advances and analysis software is updated, plant mitochondrial DNA molecular data has been increasingly used to reconstruct phylogenetic trees to infer phylogenetic processes and analyze evolutionary relationships between species [63]. In the present study, *C. retusus* was found to form a closely related cluster with the *Chionanthus* genus of Oleaceae (order Lamiales) and was genetically closer to *C. rupicola* and more closely related to *C. rupicola* than to *O. fragrans* and *F. suspensa*. This result is expected, given the relatively small number of mitochondrial genomes reported in Oleaceae and the independent genetic characteristics of mitochondria. Therefore, phylogenetic tree construction based solely on mitochondrial DNA may not accurately reflect phylogenetic relationships [47]. Overall, the use of mitochondrial DNA for phylogenetic analyses requires further testing and assessment.

Researchers can assess the assembly effect of genomes and the retention and loss of homologous genes through homology comparison by studying collinearity. In the present study, a large number of homologous collinear blocks were detected between *C. retusus* and proximal species within Oleaceae; however, these collinear blocks had shorter lengths. Furthermore, some sequences specific to *C. reusus* have been found to show blank areas because they have no homology with the other species. These nine species’ mitochondrial genomes differed in the arrangement of collinear blocks. The mitochondrial genome of *C. retusus* has undergone a large number of genome rearrangements. These nine Oleaceae species’s mitochondrial genome sequences are extremely unconserved in order of arrangement and have undergone frequent genome recombination. This may be one of the reasons for the anagenesis and diversification of mitochondrial genomes in tassel trees as a taxonomic group.

## 5. Conclusions

In this work, we studied the mitochondrial genome of ancient (>500 yr) *C. retusus* and assembled and annotated the entire mitochondrial genome of *C. retusus*, which has a single circular molecular structure with a total length of 657,640 bq and a GC content of 44.52%. A total of 37 unique protein-coding genes, 20 tRNA genes, and three rRNA genes were annotated. The *C. retusus* mitochondrial genome contained four types of repeats, and the migration of 25 fragments (total length 39,288 bp) from cpDNA transfer to mtDNA was observed. In addition, the traditional taxonomic perspective of Oleaceae was supported by phylogenetic trees based on the mitogenomes of 36 species, which helped to classify *C. retusus* scientifically. The findings reported here provide a valuable reference for further study of the genome of Oleaceae plants, with implications for the research of evolutionary biology, genetic diversity protection, and molecular breeding of species of Oleaceae. More importantly, this study is expected to provide a more in-depth basis for the protection of ancient and famous trees of Oleaceae.

## Figures and Tables

**Table 1:** Genes encoded by *C. retusus* mitochondrial genome.

Group of Genes	Name of Genes
ATP synthase	*atp*1, *atp*4, *atp*6, *atp*8, *atp*9
NADH dehydrogenase	*nad*1, *nad*2, *nad*3, *nad*4, *nad*4L, *nad*5, *nad*6, *nad*7, *nad*9
Cytochrome b	*cob*
Cytochrome c biogenesis	*ccm*B, *ccm*C, *ccm*FC, *ccm*FN
Cytochrome c oxidase	*cox*1, *cox*2, *cox*3
Maturases	*mat*R
Protein transport subunit	*mtt*B
Ribosomal protein large subunit	*rpl*2, *rpl*5, *rpl*10, *rpl*16
Ribosomal protein small subunit	*rps*3, *rps*4, *rps*7, *rps*10, *rps*12, *rps*13, *rps*14
Succinate dehydrogenase	*sdh*3, *sdh*4
Ribosome RNA	*rrn*5, *rrn*18, *rrn*26
Transfer RNA	*trn*C-GCA (×3) ^1^, *trn*D-GUC (×3), trnE-UUC (×3), *trn*F-GAA, *trnf*M-CAU, *trn*G-GCC, *trn*HGUG, *trn*I-CAU (×2), *trn*K-UUU, *trn*M-CAU (×2), *trn*N-GUU (×2), *trn*P-UGG, *trn*Q-UUG, *trn*S-CGA, *trn*S-GCU, *trn*S-GGA, *trn*S-UGA, *trn*TGGU, *trn*W-CCA (×2), *trn*Y-GUA (×3)

^1^ The gene’s number of copies is indicated by the numbers in parenthesis; for example, (×3) indicates that there are three copies.

## Data Availability

Publicly available datasets were analyzed in this study. The assembled mitogenomes were deposited in GenBank on the NCBI website (accessed on 30 July 2024, https://www.ncbi.nlm.nih.gov/), accessed under the accession numbers PQ072898.

## Supplementary Materials

The following supporting information can be downloaded at: https://www.mdpi.com/article/10.3390/genes15121523/s1, Figure S1: Potential recombination configuration of mitochondrial genome.; Table S1: Length of each node and sequencing depth.; Table S2: Relative synonymous codon usage for each amino acid in the *C. retusus* mitochondrial genome.; Table S3: Mitochondrial coding gene of *C. retusus*.
