# Supplementary material for: Mitochondrial Genome Assembly and Comparative Analysis of Chionanthus Retusus (Oleaceae)
Source: Genes (Basel). 2024 Nov 27;15(12):1523. doi: 10.3390/genes15121523 (PMC11675231; doi:10.3390/genes15121523)
Supplement: Supplementary file 1 [file genes-15-01523-s001.zip › genes-3321100-supplementary.pdf]

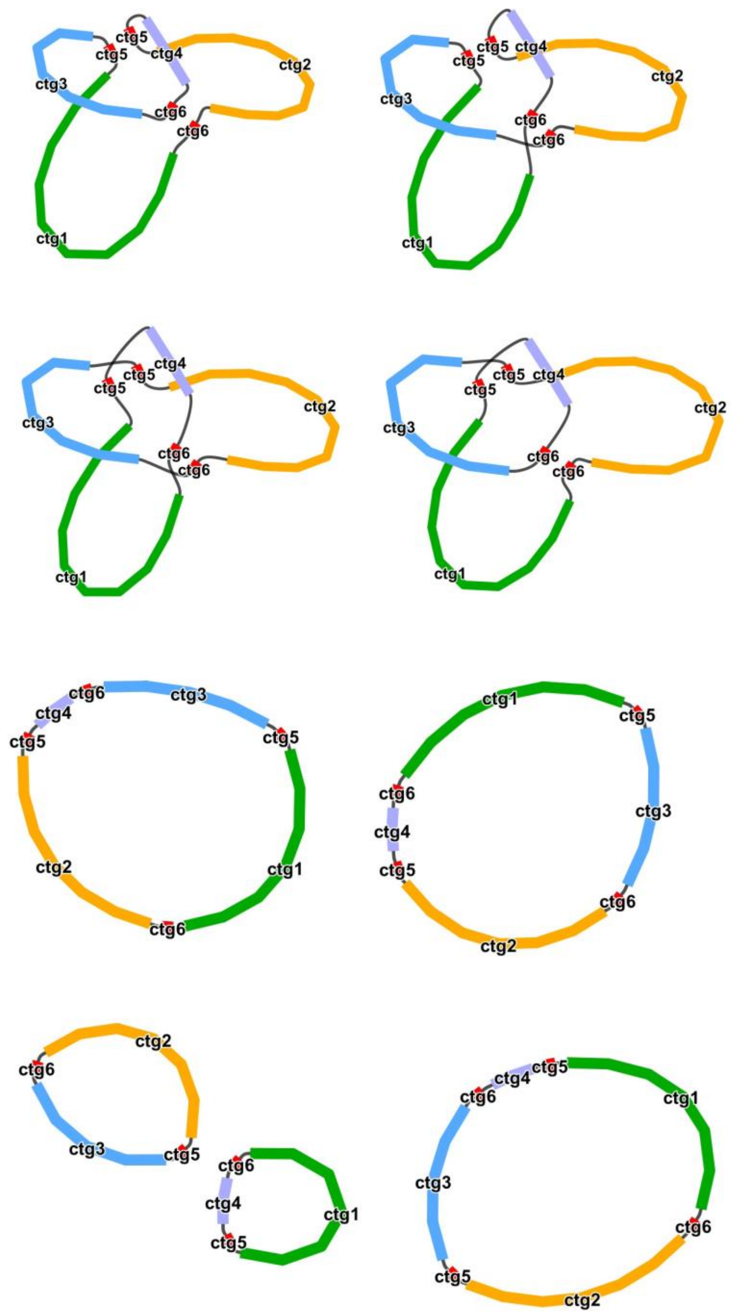

Figure S1. Potential recombination configuration of mitochondrial genome.

Table S1. Length of each node and sequencing depth.

Contig/Node	Length (bp)	Depth (×)
1	232,830	129
2	215,108	122
3	150,458	132
4	42,290	134
5	7,661	334
6	816	262

Table S2. Relative synonymous codon usage for each amino acid in the *C. retusus* mitochondrial genome.

Amino	Codon 1	Codon 2	Codon 3	Codon 4	Codon 5	Codon 6

	RSCU	RSCU	RSCU	RSCU	RSCU	RSCU
Ala	GCU<br>1.63	GCA<br>0.98	GCC<br>0.91	GCG<br>0.48		
Arg	AGA<br>1.46	CGA<br>1.35	CGU<br>1.29	AGG<br>0.73	CGG<br>0.61	CGC<br>0.57
Asn	AAU<br>1.36	AAC<br>0.64				
Asp	GAU<br>1.38	GAC<br>0.62				
Cys	UGU<br>1.2	UGC<br>0.8				
End	UAA<br>1.63	UGA<br>1.03	UAG<br>0.34			
Gln	CAA<br>1.49	CAG<br>0.51				
Glu	GAA<br>1.4	GAG<br>0.6				
Gly	GGA<br>1.45	GGU<br>1.33	GGG<br>0.69	GGC<br>0.52		
His	CAU<br>1.52	CAC<br>0.48				
Ile	AUU<br>1.32	AUA<br>0.85	AUC<br>0.83			
Leu	UUA<br>1.43	CUU<br>1.26	UUG<br>1.25	CUA<br>0.91	CUC<br>0.6	CUG<br>0.54
Lys	AAA<br>1.2	AAG<br>0.8				
Met	AUG<br>1					
Phe	UUU<br>1.16	UUC<br>0.84				
Pro	CCU<br>1.48	CCA<br>1.13	CCC<br>0.77	CCG<br>0.62		
Ser	UCU<br>1.42	UCA<br>1.16	AGU<br>1.04	UCC<br>0.95	UCG<br>0.83	AGC<br>0.6
Thr	ACU<br>1.39	ACA<br>1.07	ACC<br>0.97	ACG<br>0.58		
Trp	UGG<br>1					
Tyr	UAU<br>1.52	UAC<br>0.48				
Val	GUU<br>1.26	GUA<br>1.23	GUG<br>0.8	GUC<br>0.71		

Table S3. Mitochondrial coding gene of *C. retusus*.

	Alignment Length	Identity%	Mismatches	Gap Openings	Alignment start (chloroplast genome)	Alignment end (chloroplast genome)	Alignment start (mitochondrial genome)	Alignment end (mitochondrial genome)	MTPT annotation
1	3602	100	0	0	29064	32665	452811	456412	complete trnC-GCA;complete petN ;complete psbM ;complete trnD-GUC;complete trnY-GUA;complete trnE-UUC
2	325	100	0	0	56177	56501	149215	149539	partial atpB
3	32	100	0	0	113520	113551	541621	541590	partial ndhF
4	31	100	0	0	42729	42759	451692	451722	partial psaA
5	31	100	0	0	42729	42759	654700	654670	partial psaA
6	3571	99.972	1	0	152048	155618	467798	471368	partial ycf2 ;complete trnI-CAU;complete rpl23 ;complete rpl2 ;complete rpl2
7	4211	99.953	2	0	17777	21987	401535	397325	partial rpoC2 ;partial rpoC1
8	4073	99.951	2	0	85971	90043	471870	467798	partial rpl22 ;complete rps19 ;complete rpl2 ;complete rpl2 ;complete rpl23 ;complete trnI-CAU;partial ycf2
9	4096	99.878	4	1	41044	45139	313770	317864	partial psaB ;complete psaA ;partial ycf3
10	1052	99.81	1	1	54169	55220	146804	147854	partial trnV-UAC;complete trnM-CAU;complete atpE ;partial atpB
11	8055	99.727	17	4	29064	37115	653581	645529	complete trnC-GCA;complete petN ;complete psbM ;complete trnD-GUC;complete

									trnY-GUA;complete trnE-UUC;complete trnT-GGU;complete psbD ;complete psbC
12	2714	99.558	0	1	24585	27298	285070	282369	partial rpoC1 ;partial rpoB
13	2135	99.438	8	3	57349	59483	143814	145944	partial rbcL ;partial accD
14	931	98.926	9	1	55271	56201	568450	569379	partial atpB
15	754	98.011	4	1	23751	24504	285803	285061	partial rpoC1
16	497	95.775	13	2	16803	17298	600507	600996	partial rps2
17	71	95.775	3	0	110626	110696	567011	566941	partial trnN-GUU
18	71	95.775	3	0	131395	131465	566941	567011	partial trnN-GUU
19	85	95.294	3	1	110621	110704	48348	48264	complete trnN-GUU
20	85	95.294	3	1	131387	131470	48264	48348	complete trnN-GUU
21	80	93.75	5	0	54372	54451	519143	519064	complete trnM-CAU
22	122	89.344	9	2	72574	72691	352400	352521	partial clpP
23	191	86.387	19	7	31954	32139	21716	21904	complete trnD-GUC
24	945	85.714	91	19	46972	47907	21193	20284	complete trnS-GGA;partial rps4
25	922	83.406	87	25	66318	67207	543718	544605	complete psbJ ;complete psbL ;complete psbF ;complete psbE
